# Cervical cancer screening of underserved women in the United States: results from the National Breast and Cervical Cancer Early Detection Program, 1997–2012

**DOI:** 10.1007/s10552-015-0524-5

**Published:** 2015-03-18

**Authors:** Florence K. L. Tangka, David H. Howard, Janet Royalty, Lucinda P. Dalzell, Jacqueline Miller, Brett J. O’Hara, Susan A. Sabatino, Kristy Joseph, Kristy Kenney, Gery P. Guy, Ingrid J. Hall

**Affiliations:** 1 Division of Cancer Prevention and Control, Centers for Disease Control and Prevention (CDC), 4770 Buford Highway NE, Mailstop F-76, Atlanta, GA 30341-3717 USA; 2 Department of Health Policy and Management, Emory University, 1518 Clifton Road NE, Atlanta, GA 30322 USA; 3 Social, Economics and Household Statistics Division, US Census Bureau, Washington, DC 20233-8510 USA; 4 Division of Global Health Protection, Center for Global Health, CDC, 4770 Buford Highway NE, Mailstop E-93, Atlanta, GA 30329 USA

**Keywords:** Cervical cancer, Papanicolaou test utilization, Screening proportions, Medically underserved, National Breast and Cervical Cancer Early Detection Program

## Abstract

**Objective:**

The National Breast and Cervical Cancer Early Detection Program (NBCCEDP) provides breast and cervical cancer screens to low-income, uninsured, and underinsured women. We describe the number and proportion of women eligible for cervical cancer screening services and the proportion of eligible women screened over the period 1997–2012.

**Methods:**

Low-income, uninsured, and underinsured women aged 18–64 years who have not had a hysterectomy are eligible for cervical cancer screening through the NBCCEDP. We estimated the number of low-income, uninsured women using data from the US Census Bureau. We adjusted our estimates for hysterectomy status using the National Health Interview Survey and the Behavioral Risk Factor Surveillance System. We used data from the NBCCEDP to describe the number of women receiving NBCCEDP-funded screening and calculated the proportion of eligible women who received screening through the NBCCEDP at the national level (by age group, race/ethnicity) and at the state level by age group. We used the Medical Expenditure Panel Survey to estimate the proportion of NBCCEDP-eligible women who were screened outside the NBCCEDP and the proportion that are not screened.

**Results:**

We estimate that in 2010–2012, 705,970 women aged 18–64 years, 6.5 % (705,970 of 9.8 million) of the eligible population, received NBCCEDP-funded Pap tests. We estimate that 60.2 % of eligible women aged 18–64 years were screened outside the NBCCEDP and 33.3 % were not screened. The NBCCEDP provided 623,603 screens to women aged 40–64 years, an estimated 16.5 % of the eligible population, and 83,660 screens to women aged 18–39 years, representing an estimated 1.2 % of the eligible population. The estimated proportions of eligible women screened in each state ranged from 1.5 to 32.7 % and 5 % to 73.2 % among the 18–64 and 40–64 years age groups, respectively. Changes in the proportion of eligible women screened over the study period were nonsignificant.

**Conclusions:**

Although the program provided cervical screening to over 700,000 women between 2010 and 2012, it served a small percent of those eligible. The proportion of women screened varied substantially across age groups, racial/ethnic groups, and states. Many low-income, uninsured women are not being screened.

## Introduction

The incidence of and mortality from cervical cancer in the USA have declined over time as a result of progress in primary prevention, early-stage disease detection, and treatment [1]. However, there are still opportunities to improve the care of women with cervical cancer. In 2010, 11,918 women were diagnosed with cervical cancer, and 3,939 women died from the disease [2].

The United States Preventive Services Task Force (USPSTF) recommends screening for cervical cancer in women aged 21–65 every 3 years with Papanicolaou smear testing (Pap test) or in women aged 30–65 every 5 years with a combination of Pap test and human papillomavirus testing [3]. The *Healthy People 2020* objective for cervical cancer screening is to screen 93 % of women aged 21–65 years by the year 2020 [4]. Current screening proportions fall short of this target and, in fact, have exhibited a small but statistically significant decline from 2000–2010. As of 2010, only 83 % of women in this 21–65 years age group were up-to-date with screening. Screening proportions for some groups of women are even lower, including Asian women (75 %), and women lacking a usual source of care (65 %) or health insurance (64 %) (CDC 2012b) [5].

To reduce disparities in cervical cancer screening proportions, the US Congress established the National Breast and Cervical Cancer Early Detection Program (NBCCEDP) in 1991 to help low-income, underinsured, and uninsured women gain access to screening and diagnostic exams for breast and cervical cancer [6]. The NBCCEDP is implemented through cooperative agreements between the Centers for Disease Control and Prevention (CDC) and 67 grantees representing health departments in all 50 states, the District of Columbia (DC), 5 US territories, and 11 American Indian and Alaska Native (AIAN) tribes or tribal organizations. The grantees typically establish subcontracts with healthcare providers across the state to deliver screening services. The local healthcare providers are diverse and include local health departments, Federally Qualified Health Centers, community health centers, Indian Health Service clinics, private clinics, hospitals, and other healthcare systems.

In 2012, the NBCCEDP provided $158 million to these 67 grantees. Per congressional mandate, at least 60 % of federal funds received by the grantees must be spent on provision of clinical services. The remaining funds are used for program management, data collection, quality assurance and improvement, partnership development, professional education, public education, outreach, and evaluation. The NBCCEDP provides cervical cancer screening services to low-income, uninsured women aged 21–64 years. Estimates of NBCCEDP reach for breast cancer screening are reported elsewhere in this monograph [7]. Treatment for women diagnosed with cervical cancer through the NBCCEDP is covered by state Medicaid funding through the Breast and Cervical Cancer Treatment Act of 2000 (Public Law 106-354), the Native American Breast and Cervical Cancer Treatment Technical Amendment Act of 2001 (Public Law 107-121), and outside sources. A detailed description of the NBCCEDP and its history are available elsewhere [6, 8, 9].

Our previous report [10] was the first to describe the extent to which the NBCCEDP helped meet the cervical cancer screening needs of the underserved population in the USA. Previously, we estimated that the NBCCEDP screened 8.7 % of eligible women aged 18–64 years for cervical cancer over the period 2004–2006. The proportion of women screened varied by age group, race/ethnicity, and across states. We estimated that the NBCCEDP screened 22.6 % of eligible women aged 40–64 years, 2.3 % of eligible women aged 18–39 years, 7.3 % of eligible Hispanic women, 6.5 % of eligible non-Hispanic black women, and 9.7 % of eligible non-Hispanic white women [10]. The purpose of this study is to update the 2004–2006 analysis by Tangka et al. [10], using data from 2010 to 2012 and to describe trends in the number of women eligible and proportion of eligible women screened between 1997 and 2012. We also estimated the proportion of women screened by state and by race/ethnicity, the proportion of eligible women who received non-NBCCEDP-funded Pap tests, and the proportion of eligible women who are not screened. This report describes the extent of the nation’s only organized screening program provision of cervical cancer screening services to underserved women in the USA over time. Information from this study is crucial for understanding the reach of the NBCCEDP, identifying populations that could benefit from better access to screening services, and targeting specific interventions for hard-to-reach women.

## Methods

### Eligibility for the NBCCEDP cervical cancer screening services

We used data from the Current Population Survey Annual Social and Economic Supplement (CPS ASEC) for calendar years 1997–2012 to estimate the number of eligible women for 3-year time intervals by age group, state, and race/ethnicity. The CPS is a monthly survey conducted by the US Census Bureau for the Bureau of Labor Statistics that collects information on employment; as well as demographic information including age, family size, sex, race, and Hispanic origin. The US Census Bureau applies a probability sample to draw about 100,000 addresses (78,000 households) participating in the CPS for the CPS ASEC. Interviewed households are asked a set of supplementary questions about their health insurance coverage and income during the previous year [11]. Respondents were considered uninsured if they were not covered by any type of private or government health insurance for the entire previous year. The methods used to collect and report CPS ASEC are described elsewhere [11–14].

The NBCCEDP provides cervical cancer screening services to uninsured or underinsured low-income women aged 21–64 years (18–64 years before the USPSTF cervical cancer recommendation of 2012 [3]) with a cervix (those who have not had a hysterectomy with removal of the cervix). Underinsured women are those with limited coverage or a high deductible or co-payment for cervical cancer screening. Women with incomes 250 % of the federal poverty level (FPL) are classified as “low income” [6]. NBCCEDP grantees have the flexibility to establish their own eligibility criteria within federal guidelines. As of 2012, 31 grantees set income eligibility criteria at 250 % of FPL and 20 set income eligibility criteria at lower poverty levels (17 at 200 % FPL, two at 225 % FPL, and one at 185 % FPL). Alaska and Hawaii have higher poverty level thresholds that are not reflected in the US Census Bureau’s definition of poverty. Estimates were adjusted to approximate the FPL in those states.

The CDC prioritizes screening of women aged 40–64 years who have not been screened in the past 5 years for cervical cancer [15]. In 2012, about half of all NBCCEDP grantees restricted eligibility to women aged 40 or older. Although the majority of women 65 years and older are covered by Medicare and are not eligible for the NBCCEDP, some NBCCEDP grantees screen women age 65 years and older who are either ineligible or cannot afford the premium to enroll in Medicare part B. Our analysis includes only women aged 18–64 years. Women without a cervix are not eligible for cervical cancer screening through the NBCCEDP. The CPS ASEC does not record whether respondents have had a hysterectomy, and so we adjusted estimates of the number of eligible women downward to account for the proportion of women who have had a hysterectomy.

We used the National Health Interview Survey (NHIS) to estimate the proportion of women in the USA who have had a hysterectomy. The NHIS is the principal source of information on the health of a nationally representative sample of the civilian non-institutionalized US population. Conducted annually by CDC’s National Center for Health Statistics (NCHS), the NHIS collects health information during in-person interviews. Each year one or more supplements are included in the NHIS that focus on specific health topics. The 2005 NHIS supplement on cancer control was able to provide estimates of the national proportion of US women who have had a hysterectomy by socioeconomic group [16]. A full description of the NHIS is available online [17].

We used the BRFSS to estimate the proportion of women who have had a hysterectomy at the state level. BRFSS is a state-based telephone survey of the civilian, non-institutionalized adult population and collects information on health practices and risk behaviors. A full description of the BRFSS is available online [18]. Because of the small proportion of women without a cervix in the three age groups in each state, we used the percentages of women who had a hysterectomy for each age category, irrespective of income and insurance status, to make the adjustment at the state level. In this article, “eligible women,” refers to the women eligible for NBCCEDP-funded Pap test, “women screened” refers to women who received Pap tests within a given 3-year period, and “screened by the NBCCEDP” means screened by providers who received CDC funding from grantees of the NBCCEDP.

### The number of women screened by NBCCEDP

Information on the number of women screened by the NBCCEDP was obtained from the NBCCEDP grantees. CDC administers and collects a standardized set of data reported by grantees on all screening services funded in part or in full by the NBCCEDP, known as minimum data elements (MDEs). Service-level records are reported and include a unique patient identifier to track women receiving services over time. The MDEs include data on demographic characteristics, service dates, tests performed, test results, and outcomes. Demographic data are self-reported. The MDEs provide information on the number of women who received NBCCEDP-funded Pap tests during the study period. For the purposes of our study, we counted women screened based on their state of residency within the 50 states and DC. We classified women by age and race/ethnicity groups. More information on the components and structure of NBCCEDP and methods for collecting and reporting NBCCEDP data have been described elsewhere [6, 19, 20].

Following the previous report [10], groups were categorized for aged 18–64, 18–39, and 40–64 years. Based on Census Bureau convention, we categorized women who reported that they were of Hispanic origin as Hispanic regardless of race. We categorized the remaining women, who were non-Hispanic, into one of the following racial groups: non-Hispanic white, non-Hispanic black, non-Hispanic AIAN, and non-Hispanic Asian and Native Hawaiian and other Pacific Islander (ANHOPI). The non-Hispanic ANHOPI race category includes those who reported any combination of Asian, Native Hawaiian, or other Pacific Islander, and no other race or ethnicity. Reporting of race and Hispanic origin is optional in the NBCCEDP. Eligible population estimates were not available for 1.9 % of screened women with unknown or multiple race/ethnicity information. These women were excluded from the screening proportion calculations.

### Eligible women screened outside the NBCCEDP

We used the 2009–2011 Medical Expenditure Panel Survey (MEPS) to estimate the proportion of women receiving Pap tests in the eligible population based on the USPSTF-recommended 3-year screening interval. MEPS is a nationally representative survey of the civilian and non-institutionalized population, and is administered by the Agency for Healthcare Research and Quality. MEPS collects information including individuals’ demographics, and their health and insurance status. Using the 2011 MEPS, we calculated the proportion of women aged 18–64 years who were uninsured for the entire year, lived in households with incomes ≤250 % of the FPL, and reported having received a Pap test in the past 3 years. We applied sample weights to produce nationally representative estimates. We calculated the proportion of women screened outside the NBCCEDP by subtracting the proportion screened by the NBCCEDP from the proportion of the eligible population screened that we estimated using MEPS.

### Data analysis

We calculated the proportion of eligible women screened for 15 successive periods from 1997–2012 based on 3-year screening intervals (e.g., 1997–1999, 1998–2000, and 2010–2012). We calculated the numerator as the number of unduplicated women screened by the NBCCEDP within the 3-year interval. We chose the 3-year period to be consistent with the cervical cancer screening interval recommended by USPSTF for cervical cancer screening at the time [21]. We calculated the denominator (size of the eligible population) as an average of the 3 years within the time period.

$${\text{Screening}}\,{\text{proportion}} = \frac{{{\text{Unduplicated}}\,{\text{women}}\,{\text{screened}}\,{\text{within}}\,{\text{a}}\, 3{\text{-year}}\,{\text{interval}}}}{{ 3{\text{-year}}\,{\text{average}}\,{\text{size}}\,{\text{of}}\,{\text{the}}\,{\text{eligible}}\,{\text{population}}}}$$

Based on the number of women screened and estimates of the number of women in the 18–64, 18–39, and 40–64 age groups for all US and eligible women, we estimated the proportion of all US women and eligible women screened through the NBCCEDP. We examined the distribution of NBCCEDP cervical cancer screening by age groups, by race/ethnicity at the national level, and by age groups at the state level. State designation is based on the woman’s residence rather than the grantee providing the service. For states with tribal organizations, the state percentages include the screening data from AIAN grantees. We report the number of women eligible and the proportion of women in the population who are eligible in each state. In compliance with the NBCCEDP data use agreement, grantee- and state-specific reports of the number and proportion of eligible women screened are de-identified. We excluded two states from the analysis of variation in the proportion of women screened at the state level because they use different NBCCEDP implementation and eligibility criteria.

Estimates of the number of women eligible and the proportion of eligible women screened are based on random surveys and are thus subject to sampling error. The technique for computing confidence intervals for the estimates of the eligible women has been described previously [22]. Consistent with Census Bureau convention [14], we report 90 % confidence intervals for estimates of the eligible population and the proportion of the eligible population screened. We used *t* tests to assess the significance of differences in the proportion of women screened. Apparent differences in the trends between the various race/ethnicity groups were not tested for statistical significance. The number of women screened by NBCCEDP is an exact count, and so we do not report inferential statistics for screening totals.

## Results

### 2010–2012 National results

#### Number and percent eligible

Table 1 reports the estimated number of eligible women and the number and proportion of eligible women screened by race/ethnicity. Between 2010 and 2012, approximately 98 million women aged 18–64 years resided in the USA. We estimate that of those women, approximately 10.9 million or 11.1 % were eligible for a NBCCEDP-funded Pap test. We estimate that more women aged 18–39 years were eligible than women aged 40–64 years (7.1, 3.8 million). We estimate that although fewer Hispanic than non-Hispanic women aged 18–64 years were eligible (3.8, 7.1 million), the percentage of all Hispanic women who were eligible was significantly larger than that of non-Hispanic women (24.7 %, 8.5 %). We estimate that within the non-Hispanic eligible population, white women constituted the largest group (4.5 million). However, the percentage of all white women eligible for the NBCCEDP was smaller than that of black women (7.2 %, 14.2 %).

**Table 1:** National Breast and Cervical Cancer Early Detection Program (NBCCEDP) eligibility and screening for Cervical Cancer, by age group, race and ethnicity; 2010–2012

Race/ethnicity	US population^a^	Women eligible for NBCCEDP screening^b^				Eligible women screened for cervical cancer via NBCCEDP		
	Number (in thousands)	Number (in thousands)^c^	(90 % CI)	%^d^	(90 % CI)	Number	%^e^	(90 % CI)
*18–64*								
Total^f^	98,212	10,887	(10,714–11,061)	11.1	(10.9–11.3)	705,970	6.5	(6.4–6.6)
Non-Hispanic	82,709	7,063	(6,921–7,206)	8.5	(8.3–8.7)	503,263	7.1	(7.0–7.3)
White	62,901	4,516	(4,401–4,631)	7.2	(7.0–7.4)	321,892	7.1	(6.9–7.3)
Black	12,884	1,826	(1,754–1,898)	14.2	(13.6–14.8)	99,743	5.5	(5.2–5.7)
AIAN	713	153	(128–178)	21.5	(18.0–25.0)	34,718	22.7	(18.9–26.4)
ANHOPI	6,211	568	(520–616)	9.1	(8.3–9.9)	42,339	7.5	(6.8–8.1)
Multiracial	–	–	–	–	–	4,571	–	–
Hispanic	15,503	3,824	(3,717–3,931)	24.7	(24.0–25.4)	193,763	5.1	(4.9–5.2)
Unknown	–	–	–	–	–	8,944	–	–
*18–39*								
Total	45,432	7,107	(6,971–7,244)	15.6	(15.3–15.9)	83,660	1.2	(1.2–1.2)
Non-Hispanic	36,453	3,970	(3,870–4,070)	10.9	(10.6–11.2)	50,713	1.3	(1.2–1.3)
White	26,445	2,489	(2,410–2,568)	9.4	(9.1–9.7)	26,705	1.1	(1.0–1.1)
Black	6,379	1,066	(1,014–1,118)	16.7	(15.9–17.5)	4,939	0.5	(0.4–0.5)
AIAN	370	98	(77–119)	26.5	(20.8–32.2)	15,362	15.7	(12.3–19.0)
ANHOPI	3,259	319	(285–352)	9.8	(8.8–10.8)	2,406	0.8	(0.7–0.8)
Multiracial	–	–	–	–	–	1,301	–	–
Hispanic	8,978	2,477	(2,395–2,559)	27.6	(26.7–28.5)	31,837	1.3	(1.2–1.3)
Unknown	–	–	–	–	–	1,110	–	–
*40–64*								
Total	52,780	3,780	(3,683–3,877)	7.2	(7.0–7.4)	623,603	16.5	(16.1–16.9)
Non-Hispanic	46,256	3,093	(3,002–3,185)	6.7	(6.5–6.9)	453,246	14.7	(14.2–15.1)
White	36,456	2,027	(1,953–2,101)	5.6	(5.4–5.8)	295,613	14.6	(14.1–15.1)
Black	6,506	760	(719–800)	11.7	(11.1–12.3)	94,849	12.5	(11.8–13.2)
AIAN	343	55	(45–65)	16.1	(13.2–19.0)	19,505	35.3	(29.0–41.7)
ANHOPI	2,952	250	(225–275)	8.5	(7.7–9.3)	39,992	16.0	(14.4–17.6)
Multiracial	–	–	–	–	–	3,287	–	–
Hispanic	6,524	1,347	(1,293–1,400)	20.6	(19.8–21.4)	162,508	12.1	(11.6–12.5)
Unknown	–	–	–	–	–	–	7,849	–

*Source*: Authors’ tabulations of modified data from the US Census Bureau, Current Population Survey, 2010–2012 Annual Social and Economic Supplements, and from NBCCEDP October 2013 data. The modification of the data was the authors’ tabulations of data from 2005 National Health Interview Survey and 2005 Behavioral Risk Factor Surveillance System

*AIAN* American Indian/Alaska Native, *ANHOPI* Asian, Native Hawaiian, and Pacific Islander. Details may not sum to totals because of rounding

^a^The US population represents the Current Population Survey sample universe of the resident civilian non-institutionalized population of the USA

^b^Women eligible for NBCCEDP-funded Pap tests include those 18–64 years of age who have a cervix, are uninsured, and have low income (based on eligibility used in each state) aggregated to the nation. The number of eligible women could be underestimated because it excludes those who have health insurance but whose insurance does not cover cervical cancer screening and those who are uninsured for <1 year. See “Methods” section for details

^c^Number of eligible US women in a given age subgroups may not sum to totals across race and ethnicity categories. Hysterectomy adjustment factors were held constant for increased reliability across age groups within a given race or Hispanic-origin category and not across age groups for all races

^d^Percent of all US women in a given age, racial, and ethnic group who were eligible for NBCCEDP-funded Pap tests

^e^Percent of all US women in a given age, racial, and ethnic group who are eligible and who were provided with NBCCEDP-funded Pap tests

^f^Number of women *screened* at aged 18–39 and 40–64 years do not sum to the numbers for 18–64 years because age groups are not mutually exclusive over the 3-year period

#### Number and percent screened by the NBCCEDP

In 2012, 705,970 women aged 18–64 years received at least one NBCCEDP-funded Pap test within the prior 3 years (Table 1). Eighty-eight percent (88.3 %) were in the 40–64 years age group. We estimate that the NBCCEDP provided cervical cancer screening to approximately 0.7 % of USA women aged 18–64 years, 0.2 % of USA women aged 18–39 years, and 1.2 % of USA women aged 40–64 years. We estimate that 1.2 % of Hispanic women and 0.6 % of non-Hispanic women aged 18–64 years received a Pap test through the NBCCEDP. Among non-Hispanic women, we estimate that 0.5 % of white women and 0.8 % of black women were screened through the NBCCEDP.

We estimate that 6.5 % of eligible women were screened one or more times between 2010 and 2012 (Table 1). The proportion of eligible women who were screened varied by age group and race/ethnicity. We estimate that the proportion of eligible women aged 40–64 years screened (16.5 %) was higher than the proportion of women aged 18–39 years screened (1.2 %). Among women in the 18–64 years and 40–64 years age groups, the estimated proportion of non-Hispanic women screened (7.1 % among women aged 18–64 years and 14.7 % among women aged 40–64 years) was higher than the proportion of Hispanic women screened (5.1 and 12.1 %, respectively). Among non-Hispanics in the 18–64 years age group, the estimated proportion of eligible women screened ranged from 5.5 % among black women to 22.7 % among AIAN women. Screening patterns are similar among non-Hispanic women in the 40–64 years age group, with an estimated proportion of eligible women screened ranging from 12.5 % among black women to 35.3 % among AIAN women.

#### Percent screened from outside the NBCCEDP

**Fig. 1:**
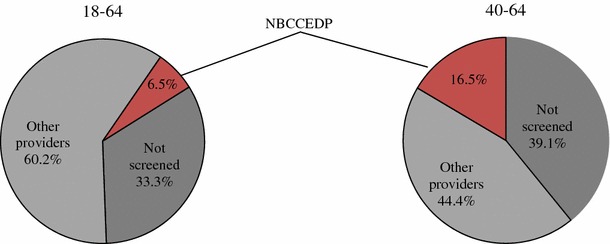
Percentage of low-income uninsured women screened for cervical cancer in the United States, 2010–2012. *Source*: Authors’ tabulations of modified data from Medical Expenditure Panel Survey 2011, US Census Bureau, Current Population Survey, 2010–2012 Annual Social and Economic Supplements and from NBCCEDP October 2013 data

Figure 1 depicts the estimated proportion of eligible women screened by the NBCCEDP, the proportion screened outside the NBCCEDP, and the proportion not screened. Comparing NBCCEDP with MEPS screening data for the NBCCEDP-eligible population, we estimated that approximately 60.2 % [90 % confidence intervals (CI): 57.5–62.7] of NBCCEDP-eligible women aged 18–64 years received a Pap test outside the NBCCEDP and the remaining 33.3 % (90 % CI: 30.8–36.0) did not receive a Pap test from any provider. We estimated that 44.4 % (90 % CI: 40.2–47.0) of women in the 40–64 years age group were screened outside the NBCCEDP and 39.1 % (90 % CI: 35.7–42.5) were not screened.

### 2010–2012 State-level results

Table 2 and “Appendix” show the estimated number and percentage of women who were eligible for NBCCEDP in each state for the 18–64, 40–64, and 18–39 years age groups. Across states, the number of eligible women ranges from about 11,000 in Vermont to about 1.5 million in California for the 18–64 years age group. The percentage of eligible women ranged from 2.5 to 17.9 % for women aged 18–64 years, and from 1.5 to 10.8 % for women aged 40–64 years.

**Table 2:** Estimated number of women eligible for cervical cancer screening in NBCCEDP, by state: 3-year averages for 2010–2012

	Poverty criterion^c^	18–64					40–64				
		US population^a^	Eligible women*				US population^a^	Eligible women^b^			
		Number^d^	Number^d^	(90 % CI)^d^	% of total^e^	(90 % CI)^d^	Number^d^	Number^d^	(90 % CI)^d^	% of total^e^	(90 % CI)^d^
USA		98,212	10,887	(10,714–11,061)	11.1	(10.9–11.3)	52,780	3,780	(3,683–3,877)	7.2	(7.0–7.4)
Alabama	200	1,535	146	(124–167)	9.5	(8.1–10.9)	870	44	(35–54)	5.1	(4.0–6.2)
Alaska	250	221	31	(27–34)	13.8	(12.0–15.6)	116	11	(9–13)	9.2	(7.6–10.8)
Arizona	250	2,011	267	(234–299)	13.3	(11.7–14.9)	1,095	103	(86–119)	9.4	(7.9–10.9)
Arkansas	200	895	122	(107–137)	13.6	(11.9–15.3)	469	38	(31–45)	8.0	(6.5–9.5)
California	200	11,975	1,480	(1,410–1,550)	12.4	(11.8–13.0)	6,141	563	(521–605)	9.2	(8.5–9.9)
Colorado	250	1,614	155	(137–173)	9.6	(8.5–10.7)	842	48	(41–54)	5.6	(4.8–6.4)
Connecticut	200	1,143	53	(43–62)	4.6	(3.8–5.4)	666	20	(15–24)	2.9	(2.2–3.6)
Delaware	250	287	20	(17–23)	7.1	(6.0–8.2)	159	6	(5–8)	4.1	(3.1–5.1)
District of Columbia	250	228	15	(12–17)	6.4	(5.3–7.5)	89	4	(3–5)	4.8	(3.6–6.0)
Florida	200	5,958	798	(744–852)	13.4	(12.5–14.3)	3,407	294	(265–324)	8.6	(7.7–9.5)
Georgia	200	3,190	427	(390–464)	13.4	(12.2–14.6)	1,706	135	(117–153)	7.9	(6.9–8.9)
Hawaii	250	417	26	(22–31)	6.3	(5.2–7.4)	225	12	(10–15)	5.4	(4.3–6.5)
Idaho	200	464	66	(58–75)	14.3	(12.4–16.2)	240	16	(12–19)	6.6	(5.2–8.0)
Illinois	250	4,034	446	(405–487)	11.1	(10.1–12.1)	2,138	166	(146–187)	7.8	(6.8–8.8)
Indiana	200	2,008	194	(166–222)	9.7	(8.3–11.1)	1,056	66	(50–82)	6.2	(4.7–7.7)
Iowa	250	950	78	(68–88)	8.2	(7.2–9.2)	523	27	(23–31)	5.2	(4.4–6.0)
Kansas	225	854	82	(71–94)	9.7	(8.4–11.0)	434	20	(16–25)	4.7	(3.7–5.7)
Kentucky	250	1,387	180	(159–200)	13.0	(11.5–14.5)	753	57	(48–66)	7.5	(6.3–8.7)
Louisiana	250	1,416	236	(208–264)	16.7	(14.7–18.7)	751	78	(65–91)	10.4	(8.6–12.2)
Maine	250	432	28	(23–32)	6.4	(5.3–7.5)	258	13	(10–16)	5.1	(4.0–6.2)
Maryland	250	1,927	160	(141–178)	8.3	(7.3–9.3)	1,061	58	(49–68)	5.5	(4.6–6.4)
Massachusetts	250	2,143	54	(39–69)	2.5	(1.8–3.2)	1,177	18	(11–25)	1.5	(0.9–2.1)
Michigan	250	3,084	283	(254–312)	9.2	(8.3–10.1)	1,723	112	(96–129)	6.5	(5.6–7.4)
Minnesota	250	1,643	100	(86–114)	6.1	(5.3–6.9)	866	28	(22–34)	3.2	(2.5–3.9)
Mississippi	250	907	121	(106–136)	13.3	(11.7–14.9)	486	34	(27–41)	7.0	(5.6–8.4)
Missouri	200	1,853	184	(160–209)	10.0	(8.7–11.3)	1,029	59	(47–72)	5.8	(4.6–7.0)
Montana	200	300	41	(34–47)	13.5	(11.4–15.6)	161	13	(10–16)	8.2	(6.4–10.0)
Nebraska	225	564	50	(43–58)	8.9	(7.6–10.2)	299	14	(11–17)	4.7	(3.8–5.6)
Nevada	250	848	143	(129–157)	16.9	(15.3–18.5)	441	46	(39–53)	10.5	(8.9–12.1)
New Hampshire	250	427	32	(28–36)	7.5	(6.5–8.5)	255	13	(11–16)	5.3	(4.4–6.2)
New Jersey	250	2,779	297	(260–334)	10.7	(9.4–12.0)	1,548	124	(106–142)	8.0	(6.8–9.2)
New Mexico	250	634	114	(100–127)	17.9	(15.8–20.0)	351	38	(31–45)	10.8	(8.9–12.7)
New York	250	6,312	569	(521–616)	9.0	(8.2–9.8)	3,347	207	(183–232)	6.2	(5.5–6.9)
North Carolina	250	3,039	400	(362–439)	13.2	(11.9–14.5)	1,652	135	(118–153)	8.2	(7.1–9.3)
North Dakota	200	213	15	(12–17)	6.8	(5.6–8.0)	111	5	(4–6)	4.5	(3.5–5.5)
Ohio	200	3,566	310	(277–343)	8.7	(7.8–9.6)	1,964	123	(104–143)	6.3	(5.3–7.3)
Oklahoma	185	1,145	125	(109–141)	10.9	(9.5–12.3)	606	38	(30–46)	6.3	(4.9–7.7)
Oregon	250	1,243	150	(132–168)	12.0	(10.6–13.4)	681	46	(37–55)	6.7	(5.4–8.0)
Pennsylvania	250	4,043	341	(305–377)	8.4	(7.5–9.3)	2,276	134	(116–152)	5.9	(5.1–6.7)
Rhode Island	250	340	29	(24–33)	8.4	(7.1–9.7)	188	12	(10–14)	6.3	(5.2–7.4)
South Carolina	200	1,503	172	(152–193)	11.5	(10.1–12.9)	812	55	(45–65)	6.8	(5.6–8.0)
South Dakota	200	247	24	(21–28)	9.8	(8.4–11.2)	127	6	(5–8)	5.0	(3.8–6.2)
Tennessee	250	2,030	201	(176–226)	9.9	(8.7–11.1)	1,109	81	(67–94)	7.3	(6.1–8.5)
Texas	200	8,068	1,371	(1,307–1,436)	17.0	(16.2–17.8)	4,066	396	(365–426)	9.7	(8.9–10.5)
Utah	250	826	87	(73–101)	10.5	(8.8–12.2)	353	20	(15–25)	5.7	(4.2–7.2)
Vermont	250	204	11	(9–13)	5.5	(4.5–6.5)	122	3	(2–5)	2.9	(2.1–3.7)
Virginia	200	2,606	217	(192–242)	8.3	(7.3–9.3)	1,424	83	(70–95)	5.8	(4.9–6.7)
Washington	250	2,175	242	(214–270)	11.1	(9.8–12.4)	1,175	83	(68–97)	7.0	(5.8–8.2)
West Virginia	200	595	61	(52–69)	10.2	(8.7–11.7)	355	23	(19–27)	6.5	(5.3–7.7)
Wisconsin	250	1,755	116	(96–137)	6.6	(5.4–7.8)	981	43	(32–54)	4.4	(3.3–5.5)
Wyoming	250	175	19	(16–22)	10.9	(9.4–12.4)	96	6	(5–8)	6.6	(5.3–7.9)

*Source*: Authors’ tabulations of modified data from the US Census Bureau, Current Population Survey, 2010–2012 Annual Social and Economic Supplements. The modification of the data was the authors’ tabulations of data from 2005 National Health Interview Survey and 2005 Behavioral Risk Factor Surveillance System

Details may not sum to totals because of rounding

^a^The US population represents the Current Population Survey sample universe which consists of the resident civilian non-institutionalized population of the USA

^b^Women eligible for NBCCEDP-funded Pap tests include those 18–64 years of age who have a cervix, are uninsured, and have low income (based on eligibility used in each state) aggregated to the nation. The number of eligible women could be underestimated because it excludes those who have health insurance but whose insurance does not cover cervical cancer screening and those who are uninsured for <1 year. See “Methods” section for details

^c^30 states and District of Columbia set income eligibility at 250 % of poverty, 18 states at 200 % of poverty, 2 states at 225 %, and 1 state at 185 % of poverty. The estimated number of women for the USA is based on the eligibility criteria used in each state

^d^Number in thousands

^e^Eligible women as percentage of all women in a given age in that state

**Fig. 2:**
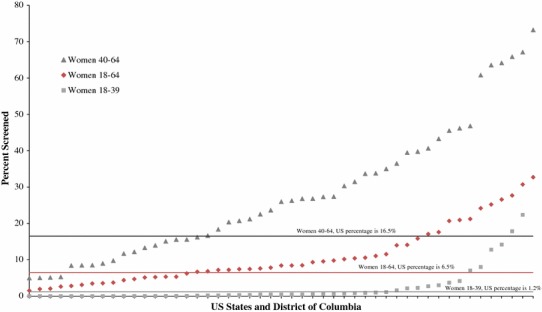
Percent of NBCCEDP-eligible women screened for cervical cancer screening by state and DC compared to national average, 2010–2012. *Source*: Authors’ tabulations of modified data from the US Census Bureau, Current Population Survey, 2010–2012 Annual Social and Economic Supplements, and from NBCCEDP October 2013 data. The modification of the data was the authors’ tabulations of data from 2005 National Health Interview Survey and 2005 Behavioral Risk Factor Surveillance System. *Notes*: The symbols show the percentage of eligible women screened by each state and District of Columbia. Two states that use different eligibility/implementation criteria are not included. Data points for each age group are sorted by percentage of eligible women screened. The proportion of NBCCEDP-eligible women screened by the NBCCEDP across the US is 1.2 % aged 18–39, 6.5 % aged 18–64, and 16.5 aged 40–64

Figure 2 depicts the estimated proportion of eligible women screened by state. The proportion of eligible women who were screened from 2010 to 2012 through the NBCCEDP varied across states. The estimated proportions of women screened by state state-level screening proportion ranged from 1.5 to 32.7 %, 0.001 to 22.4 %, and 5.0 % to 73.2 % in the 18–64, 18–39, and 40–64 years age groups, respectively. The median estimated proportion of women aged 18–64 and 40–64 years screened was 10.4 and 31.5 %, respectively. The 25th and 75th percentiles for the 18–64 years age group are 5.2 and 14.0 %, respectively. The equivalent figures for women in the 40–64 years age group are 14.1 and 39.5 %.

### Trends, 1997–2012 national results

**Fig. 3:**
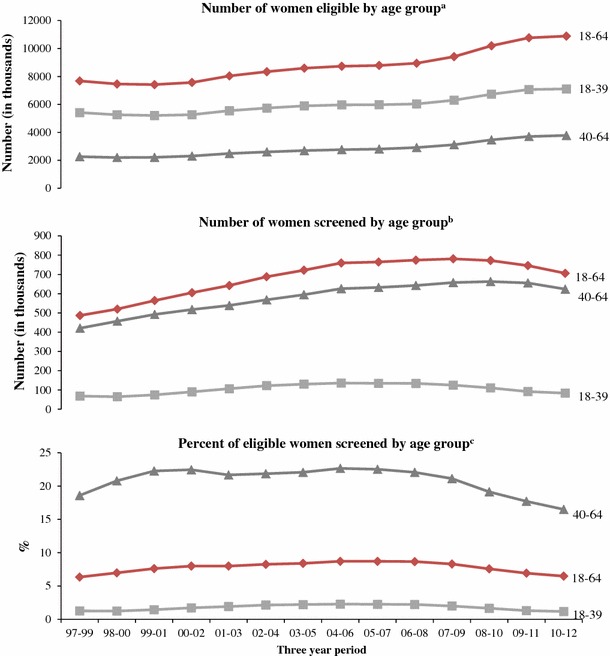
Trends in NBCCEDP-eligible population and reach for cervical cancer screening by age group. ^a^Women eligible for NBCCEDP-funded Pap tests include those 18–64 years of age who have a cervix, are uninsured, and have low income (based on eligibility used in each state) aggregated to the nation. The number of eligible women could be underestimated because it excludes those who have health insurance, but whose insurance does not cover cervical cancer screening and those who are uninsured for <1 year. See “Methods” section for details. ^b^Percent of all US women in a given age group who were eligible for NBCCEDP-funded Pap tests. ^c^Percent of all US women in a given age group who are eligible and who were provided with NBCCEDP-funded Pap tests. *Source*: Authors’ tabulations of modified data from the US Census Bureau, Current Population Survey, 2010–2012 Annual Social and Economic Supplements, and from NBCCEDP October 2013 data. The modification of the data was the authors’ tabulations of data from 2005 National Health Interview Survey and 2005 Behavioral Risk Factor Surveillance System

Tables 3 and Fig. 3 report the number of eligible women and the number and proportion of women screened by age group and period. The estimated number of eligible women aged 18–64 years increased by 3.5 million from 7.4 million in 1999–2001 to 10.9 million in 2010–2012. Over the same period, the estimated number of eligible women aged 18–39 years increased by 1.9 million from 5.2 million to 7.1 million. Meanwhile, the estimated number of eligible women aged 40–64 years increased by 1.6 million from 2.2 million to 3.8 million.

**Table 3:** NBCCEDP trends in the number of women eligible and the number and percent of women screened for cervical cancer

Year	Women eligible for NBCCEDP screening		Eligible women screened for cervical cancer via NBCCEDP		
	Number (in thousands)	(90 % CI)	Number (in thousands)	Percent of eligible screened	(90 % CI)
*18–64*					
1997–1999	7,681	(7,373–7,989)	487	6.3	(6.1–6.6)
1998–2000	7,458	(7,178–7,738)	520	7.0	(6.7–7.2)
1999–2001	7,417	(7,172–7,661)	564	7.6	(7.4–7.9)
2000–2002	7,570	(7,350–7,789)	605	8.0	(7.8–8.2)
2001–2003	8,039	(7,813–8,264)	643	8.0	(7.8–8.2)
2002–2004	8,341	(8,130–8,552)	688	8.2	(8.0–8.5)
2003–2005	8,594	(8,414–8,774)	722	8.4	(8.2–8.6)
2004–2006	8,730	(8,579–8,882)	760	8.7	(8.5–8.9)
2005–2007	8,787	(8,639–8,934)	764	8.7	(8.6–8.8)
2006–2008	8,945	(8,796–9,094)	774	8.7	(8.5–8.8)
2007–2009	9,415	(9,257–9,573)	781	8.3	(8.2–8.4)
2008–2010	10,196	(10,023–10,369)	772	7.6	(7.4–7.7)
2009–2011	10,768	(10,588–10,947)	746	6.9	(6.8–7.0)
2010–2012	10,887	(10,714–11,061)	706	6.5	(6.4–6.6)
Change	3,207		219	0.14	
*18–39*					
1997–1999	5,416	(5,133–5,698)	68	1.3	(1.2–1.3)
1998–2000	5,258	(5,001–5,514)	65	1.2	(1.2–1.3)
1999–2001	5,206	(4,983–5,429)	75	1.4	(1.4–1.5)
2000–2002	5,264	(5,066–5,463)	90	1.7	(1.7–1.8)
2001–2003	5,552	(5,348–5,755)	107	1.9	(1.8–2.0)
2002–2004	5,740	(5,557–5,924)	123	2.1	(2.1–2.2)
2003–2005	5,899	(5,746–6,051)	130	2.2	(2.2–2.3)
2004–2006	5,967	(5,845–6,088)	136	2.3	(2.2–2.3)
2005–2007	5,978	(5,858–6,099)	134	2.3	(2.2–2.3)
2006–2008	6,033	(5,916–6,149)	134	2.2	(2.2–2.3)
2007–2009	6,301	(6,180–6,423)	125	2.0	(2.0–2.0)
2008–2010	6,731	(6,604–6,858)	111	1.7	(1.6–1.7)
2009–2011	7,064	(6,930–7,198)	92	1.3	(1.3–1.3)
2010–2012	7,107	(6,971–7,244)	84	1.2	(1.2–1.2)
Change	1,692		15	−0.1	
*40–64*					
1997–1999	2,265	(2,103–2,428)	421	18.6	(17.3–19.9)
1998–2000	2,200	(2,052–2,348)	457	20.8	(19.4–22.2)
1999–2001	2,211	(2,081–2,341)	492	22.3	(21.0–23.6)
2000–2002	2,305	(2,188–2,423)	518	22.5	(21.3–23.6)
2001–2003	2,487	(2,365–2,609)	539	21.7	(20.6–22.7)
2002–2004	2,600	(2,487–2,714)	568	21.9	(20.9–22.8)
2003–2005	2,695	(2,598–2,792)	595	22.1	(21.3–22.9)
2004–2006	2,764	(2,682–2,845)	626	22.7	(22.0–23.3)
2005–2007	2,808	(2,727–2,889)	633	22.5	(21.9–23.2)
2006–2008	2,912	(2,830–2,994)	643	22.1	(21.5–22.7)
2007–2009	3,114	(3,027–3,201)	658	21.1	(20.5–21.7)
2008–2010	3,465	(3,370–3,561)	663	19.1	(18.6–19.7)
2009–2011	3,703	(3,603–3,804)	656	17.7	(17.2–18.2)
2010–2012	3,780	(3,683–3,877)	624	16.5	(16.1–16.9)
Change	1,515		203	−2.1	

*Source*: Authors’ tabulations of modified data from the US Census Bureau, Current Population Survey, 2010–2012 Annual Social and Economic Supplements, and from NBCCEDP October 2013 data. The modification of the data was the authors’ tabulations of data from 2005 National Health Interview Survey and 2005 Behavioral Risk Factor Surveillance System

Details may not sum to totals because of rounding

The number of women aged 18–64 years screened increased by 142,000 (from 564,000 to 706,000), with women aged 40–64 years accounting for most of the increase (132,000). Trends in the number eligible and number screened were similar among racial and ethnic groups (data not shown).

Increases in the number of women eligible outpaced the number of women screened, leading to a statistically significant decline in the proportion of women screened between 1999–2001 and 2010–2012. The estimated proportion of eligible women age 18–64 years who were screened fell from 7.6 % in 1999–2001 to 6.5 % in 2010–2012. The estimated proportion of eligible women aged 18–39 years who were screened decreased slightly from 1.4 % in 1999–2001 to 1.2 % in 2010–2012. The estimated proportion of eligible women aged 40–64 years who were screened decreased from 22.3 % in 1999–2001 to 16.5 % in 2011–2012.

**Fig. 4:**
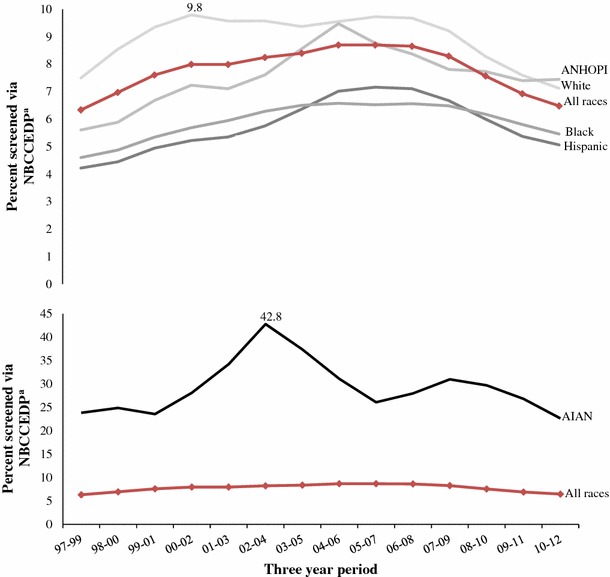
NBCCEDP trends in the percent of eligible women screened for cervical cancer, aged 18–64, by race and ethnicity. ^a^Women eligible for NBCCEDP-funded Pap tests include those 18–64 years of age who have a cervix, are uninsured, and have low income (based on eligibility criteria used in each state) aggregated to the nation. The number of eligible women could be underestimated because it excludes those who have health insurance, but whose insurance does not cover cervical cancer screening and those who are uninsured for <1 year. See “Methods” section for details. ^a^ ^b^Percent of all US women aged 18–64 who were eligible for NBCCEDP-funded Pap tests and who were provided with NBCCEDP-funded Pap tests. *Source*: Authors’ tabulations of modified data from the US Census Bureau, Current Population Survey, 2010–2012 Annual Social and Economic Supplements, and from NBCCEDP October 2013 data. The modification of the data was the authors’ tabulations of data from 2005 National Health Interview Survey and 2005 Behavioral Risk Factor Surveillance System. *Notes*: *AIAN* American Indian or Alaska Native; *ANHOPI* Asian American, Native Hawaiian, or Pacific Islander. Data are presented in two graphs because of differences in scale. Highest points are marked to point out scale

Figure 4 shows trends in the estimated proportion of eligible women aged 18–64 years screened by race/ethnicity. Estimates of changes in the proportion of women screened over the study period for black [from 4.6 % (90 % CI: 4.2–5.0 %) to 5.5 % (90 % CI: 5.3–5.7 %)], ANHOPI [from 5.6 % (90 % CI: 4.5–6.7 %) to 7.5 % (90 % CI: 6.9–8.1 %)], and Hispanic [from 4.2 % (90 % CI: 3.9–4.5 %) to 5.1 % (90 % CI: 5.0–5.2 %)] were significant at the 1 % level. The estimate of the change in the proportion of white women screened over the study period [from 7.5 % (90 % CI: 7.1–7.9 %) to 7.1 % (90 % CI: 6.9–7.3 %)] was not significant.

## Discussion

We estimate that the NBCCEDP screened approximately 7 % of the eligible population during the period 2010–2012. More than half of the eligible women screened were racial/ethnic minorities, consistent with the NBCCEDP’s goal of reducing disparities. We estimate that 60.2 % of the eligible women aged 18–64 years were screened outside the NBCCEDP, leaving approximately 33.3 % of the eligible women not screened within a 3-year period. Previously, we reported that from 2004 to 2006, 9 % of the eligible population aged 18–64 received a Pap test from the NBCCEDP, while 56.2 % were screened by other providers and 34.8 % were not screened [10]. The Government Accountability Office (GAO) conducted a separate study to examine, among other things, the NBCCEDP’s screening of eligible women [15]. GAO used the MEPS as the source of data to estimate the number of women eligible for the NBCCEDP, from 2004 to 2006. GAO findings were similar to our previous findings (2004–2006). GAO estimated that from 2004 to 2006, 9 % of eligible women were screened by the NBCCEDP, 59 % by other providers, and 33 % were not screened [15]. The decline in the proportion of eligible women screened is largely due to increases in the number of women eligible. Declines in the proportion screened occurred across all race/ethnicity groups. The data in this paper provide vital information for planning, monitoring, and evaluating the only nationally organized screening program in the USA.

Similar to previous findings, the number and percentages of eligible women screened varied widely by age, race/ethnicity [10, 15], and state of residence [10]. The NBCCEDP was most successful in meeting the needs of women aged 40–64 years, although the percent of women eligible for services is higher in the 18–39 years age group. Some grantees prioritize serving women aged 40–64 years to focus outreach efforts to women for both breast and cervical cancer screenings.

The percentage of the eligible women screened by the NBCCEDP is small. A large number of federally funded community health centers, hospitals, family planning clinics, and voluntary associations provide cervical cancer screening services to underserved women outside of the NBCCEDP. Although most eligible women receive cervical screening services from other providers, 33.3 % of eligible women aged 18–64 years were not screened. This figure is consistent with other recent estimates of screening among uninsured women [5]. Possible reasons why more eligible women do not receive cervical cancer screening include fear of painful procedures, fear of having cancer, lack of insurance, high deductibles and co-payments, lack of a usual source of care, lack of knowledge about screening or recommended screening intervals, lack of transportation, and lack of nearby providers [10, 15, 23–28]. Among women using the NBCCEDP services, low education level and foreign-born status were associated with not returning for repeat screening, suggesting that low education and factors associated with foreign-born status are barriers to the use of the NBCCEDP [29]. A large proportion of eligible women do not know about the NBCCEDP [30].

Variation in screening rates across states could be explained by differences in CDC funding levels, eligibility criteria, availability of other resources, clinical costs, grantee infrastructure for management and service delivery, recruitment strategies, and the number of eligible women [10, 15]. As to be expected, eligibility rates tend to be higher in states with lower average incomes. Local characteristics such as the average cost of service delivery, size of the state population, percentage of eligible women, and the percentage of the population that resides in an urban area affect screening proportions [31]. Grantees receive varying levels of funding from the CDC, state government, and other sources. It is likely that this may influence the number of women served.

In 2010–2012, both the number of women eligible for the NBCCEDP and the number of women screened through the NBCCEDP increased, in comparison with 1999–2001. However, increases in the number of women eligible outpaced the number of women screened, resulting in a decrease in the proportion of women screened between 1999–2001 and 2010–2012. The decline in the proportion of eligible women screened is largely due to increases in the number of eligible women. Unemployment rates, which increase during and shortly after recession periods (March 2001–November 2001 and December 2007–June 2009), increased the number of people in poverty, hence the number of women eligible for the NBCCEDP. In 1999, there were 32.3 million people in poverty compared with 46.5 million people in 2012. The poverty rate increased by 3.2 percentage points, from 11.8 % in 1999 to 15.0 % in 2012 [11, 32].

Our study is subject to a number of limitations. First, our study may underestimate the number of women who are eligible for the NBCCEDP. Women who are underinsured (those whose insurance does not cover preventive services or those who have high co-payments) and are eligible for the NBCCEDP are not included in the CPS ASEC uninsured estimates and thus are not included in the denominators of the screening proportions. The CPS ASEC measures the number of women who are insured, but not the number who are *under*insured. No general definition of being underinsured is available, and the number of low-income, underinsured women in the population is unknown. This could result in an overestimate of the screening proportions. Estimates are also subject to recall bias. The CPS ASEC uses annual retrospective questions, and respondents may have difficulty recalling information about insurance coverage [33]. Considering only women uninsured for the whole year excludes women who are uninsured for only a part of the year and would be eligible for the NBCCEDP for the period that they were uninsured. Our inability to define the race and ethnicity of some women in the study could result in an underestimate of the screening proportion for any given race or ethnic group. Analyses that further stratify the data (by age group at the state level and by age group for individual race/ethnic groups) were impossible due to small sample sizes. Dalzell et al. [12] describe three US Census Bureau’s data sources for estimating the NBCCEDP-eligible population.

Second, we used BRFSS and NHIS data to adjust the estimates of the eligible population derived from the CPS ASEC for the proportion of women who have had a hysterectomy. Using data from various sources may introduce some errors in the estimates because the questionnaires, data collection methods, and sampling methods are different. Also, in these data sources, it is not possible to distinguish between women who had a partial hysterectomy instead of a total hysterectomy.

Third, because BRFSS was administered by landline telephones only during the year used in this analysis, less affluent groups, such as low-income uninsured women, may be underrepresented because they are less likely to have landline telephone service [34, 35]. In contrast, NHIS is conducted primarily by in-person interview. Last, the unit of analysis for this study is the state and not the grantee, as standardized estimates of eligible populations are not available for NBCCEDP grantees that are tribal organizations and US territories.

In 2010, *Healthy People 2020* set the objective of increasing the proportion of women aged 21–65 years who receive a Pap test within a 3-year period to 93 % [36]. Although cervical cancer screening proportions have increased over time, screening proportions among the uninsured still lag far behind those among women with private or public health insurance [5]. The Affordable Care Act (ACA) should increase access to cervical cancer screening services for many low-income, underserved women by making health insurance more available and by eliminating cost-sharing for cervical cancer screening. Additional studies will be needed to assess the impact of ACA implementation on screening uptake. There are many factors other than access to insurance that serve as barriers for underserved women to receive cancer screening [10, 15, 23–26, 28–30]. CDC, through the NBCCEDP, funds grantees to recruit women, address these barriers and improve access to screening and diagnostic services. Since the state grantees reach underserved women, the NBCCEDP provides a unique opportunity to reduce disparities in cervical cancer and increase the proportion of women screened among the underserved population. Although the number of women screened by NBCCEDP has increased since 1997, a large share of NBCCEDP-eligible women did not receive recommended Pap tests. Results of this study indicate there continues to be an unmet need for screening services for underserved populations.

## Appendix

See Table 4.

**Table 4:** Estimated number of women, aged 18–39, eligible for cervical cancer screening in NBCCEDP, by state: 3-year averages for 2010–2012

	Poverty criterion^c^	US population^a^	Eligible women^b^			
		Number (in thousands)	Number (in thousands)	90 % CI (in thousands)	% of total^d^	90 % CI (%)
*18*–*39*						
USA		45,432	7,107	(6,971–7,244)	15.6	(15.3–15.9)
Alabama	200	665	101	(84–119)	15.2	(12.5–17.9)
Alaska	250	105	20	(17–23)	18.9	(15.8–22.0)
Arizona	250	916	164	(138–190)	17.9	(15.0–20.8)
Arkansas	200	426	84	(72–97)	19.8	(16.9–22.7)
California	200	5,834	917	(865–969)	15.7	(14.8–16.6)
Colorado	250	772	107	(92–123)	13.9	(11.9–15.9)
Connecticut	200	478	33	(26–40)	6.9	(5.4–8.4)
Delaware	250	128	14	(11–16)	10.7	(8.8–12.6)
District of Columbia	250	139	10	(8–12)	7.4	(5.9–8.9)
Florida	200	2,550	503	(461–545)	19.7	(18.0–21.4)
Georgia	200	1,484	292	(262–323)	19.7	(17.7–21.7)
Hawaii	250	192	14	(11–18)	7.3	(5.5–9.1)
Idaho	200	224	51	(43–58)	22.6	(19.2–26.0)
Illinois	250	1,896	280	(246–313)	14.8	(13.0–16.6)
Indiana	200	952	128	(107–149)	13.5	(11.3–15.7)
Iowa	250	427	51	(43–59)	12.0	(10.1–13.9)
Kansas	225	420	62	(52–72)	14.8	(12.4–17.2)
Kentucky	250	634	123	(106–140)	19.4	(16.7–22.1)
Louisiana	250	666	158	(135–181)	23.8	(20.3–27.3)
Maine	250	174	15	(11–18)	8.3	(6.3–10.3)
Maryland	250	866	101	(86–117)	11.7	(9.9–13.5)
Massachusetts	250	966	36	(24–48)	3.7	(2.5–4.9)
Michigan	250	1,361	171	(148–193)	12.5	(10.9–14.1)
Minnesota	250	776	72	(60–84)	9.3	(7.8–10.8)
Mississippi	250	420	87	(74–99)	20.6	(17.6–23.6)
Missouri	200	825	125	(105–145)	15.2	(12.8–17.6)
Montana	200	139	28	(22–33)	19.8	(16.0–23.6)
Nebraska	225	265	36	(30–43)	13.7	(11.2–16.2)
Nevada	250	407	97	(85–108)	23.8	(21.0–26.6)
New Hampshire	250	172	19	(15–22)	10.8	(8.9–12.7)
New Jersey	250	1,231	173	(143–203)	14.0	(11.6–16.4)
New Mexico	250	283	76	(65–86)	26.8	(23.0–30.6)
New York	250	2,965	362	(323–400)	12.2	(10.9–13.5)
North Carolina	250	1,387	265	(233–298)	19.1	(16.8–21.4)
North Dakota	200	102	10	(7–12)	9.4	(7.2–11.6)
Ohio	200	1,602	186	(162–211)	11.6	(10.1–13.1)
Oklahoma	185	539	87	(74–100)	16.2	(13.8–18.6)
Oregon	250	562	104	(89–118)	18.5	(15.9–21.1)
Pennsylvania	250	1,767	207	(177–236)	11.7	(10.0–13.4)
Rhode Island	250	152	17	(13–20)	11.1	(8.8–13.4)
South Carolina	200	691	117	(100–134)	17.0	(14.5–19.5)
South Dakota	200	119	18	(15–21)	14.8	(12.3–17.3)
Tennessee	250	921	120	(100–140)	13.0	(10.9–15.1)
Texas	200	4,002	976	(922–1,030)	24.4	(23.1–25.7)
Utah	250	473	67	(55–79)	14.2	(11.6–16.8)
Vermont	250	82	8	(6–9)	9.4	(7.4–11.4)
Virginia	200	1,182	135	(114–155)	11.4	(9.7–13.1)
Washington	250	1,001	159	(137–182)	15.9	(13.7–18.1)
West Virginia	200	240	38	(31–45)	15.7	(12.7–18.7)
Wisconsin	250	774	73	(57–89)	9.5	(7.4–11.6)
Wyoming	250	79	13	(11–15)	16.0	(13.3–18.7)

*Source*: Authors’ tabulations of modified data from the US Census Bureau, Current Population Survey, 2010–2012 Annual Social and Economic Supplements. The modification of the data was the authors’ tabulations of data from 2005 National Health Interview Survey and 2005 Behavioral Risk Factor Surveillance System

Details may not sum to totals because of rounding

^a^The US population represents the Current Population Survey sample universe which consists of the resident civilian non-institutionalized population of the USA

^b^Women eligible for NBCCEDP–funded Pap tests include those 18–39 years of age who have a cervix, are uninsured, and have low income (based on eligibility criteria used in each state) aggregated to the nation. The number of eligible women could be underestimated because it excludes those who have health insurance but whose insurance does not cover cervical cancer screening and those who are uninsured for <1 year. See “Methods” section for details

^c^30 states and District of Columbia set income eligibility at 250 % of poverty, 18 states at 200 % of poverty, 2 states at 225 %, and 1 state at 185 % of poverty. The estimated women for the USA are based on the eligibility criteria used in each state

^d^Eligible women as percentage of all women in a given age in that state

## References

[1] Pierce Campbell CM, Menezes LJ, Paskett ED, Giuliano AR (2012). Prevention of invasive cervical cancer in the United States: past, present, and future. Cancer Epidemiol Biomarkers Prev.

[2] U.S. Cancer Statistics Working Group (2013) United States Cancer Statistics: 1999–2010 incidence and mortality web-based report. Atlanta: U.S. Department of Health and Human Services, Centers for Disease Control and Health Promotion, and National Cancer Institute (cited 2014 June 2). www.cdc.gov/uscs

[3] U.S. Preventive Services Task Force (2012) Screening for cervical cancer: clinical summary of U.S. Preventive Services Task Force recommendation. AHRQ Publication No. 11-05156-EF-3 (cited 2014 June 6). http://www.uspreventiveservicestaskforce.org/uspstf11/cervcancer/cervcancersum.htm

[4] U.S. Department of Health and Human Services. *Healthy people 2020* (cited 2014 June 6). http://www.healthypeople.gov/2020/topicsobjectives2020/objectiveslist.aspx?topicId=5

[5] CDC (2012). Cancer screening—United States, 2010. MMWR.

[6] CDC. National Breast and Cervical Cancer Early Detection Program (cited 2014 April 19). http://www.cdc.gov/cancer/nbccedp/about.htm

[7] Howard D, Tangka FKL, Royalty J, Danzell LP, Miller J, O’Hara B, Joseph K, Kenney K, Guy G, Hall IJ (2015) Breast cancer screening of underserved women in the United States: results from the National Breast and Cervical Cancer Early Detection Program, 1998–2012. Cancer Causes Control (forthcoming, this supplement) 10.1007/s10552-015-0553-0 PMC4748380 25779379

[8] Lantz P, Mullen J (2015) Overview of the NBCCEDP (forthcoming, this supplement)

[9] Lee NC, Wong FL, Jamison PM (2014). Implementation of the National Breast and Cervical Cancer Early Detection Program: the beginning. Cancer.

[10] Tangka FKL, O’Hara B, Gardner JG, Turner J, Royalty J (2010). Meeting the cervical cancer screening needs of underserved women: the National Breast and Cervical Cancer Early Detection Program, 2004–2006. Cancer Causes Control.

[11] DeNavas-Walt C, Proctor BD, Smith J (2013). U.S. Census Bureau, current population reports, series P60–245, income, poverty, and health insurance coverage in the United States 2012.

[12] Dalzell LP, Tangka FKL, Powers DS, Holmes, O’Hara BJ, Kristy J, Janet R (2015) Data sources to identify low income, uninsured populations: application to public health—National Breast and Cervical Early Detection Program (forthcoming, this supplement)

[13] U.S. Census Bureau (2006) Current population survey: design and methodology. Technical paper 66 (cited 2014 April 21). http://www.census.gov/prod/2006pubs/tp-66.pdf

[14] U.S. Census Bureau (2012) Source and accuracy of estimates for income, poverty, and health insurance coverage in the United States (cited 2014 June 8). http://www.census.gov/hhes/www/p60_245sa.pdf

[15] United States Government Accountability Office (2009) Report to congressional requesters. MEDICAID. Source of screening affect women’s eligibility for coverage of breast and cervical cancer treatment in some states. GAO-09-384 (cited 2014 June 2). http://www.gao.gov/new.items/d09384.pdf

[16] National Center for Health Statistics (2006) Data file documentation, National Health Interview Survey, 2005 (machine-readable data file and documentation). Hyattsville: U.S. Department of Health and Human Services (cited June 6 2014). ftp://cdc.gov/pub/Health_Statistics/NCHS/Dataset_Documentation/NHIS/2005/srvydesc.pdf

[17] CDC (2014) About the National Health Interview Survey (cited 2014 April 21). http://www.cdc.gov/nchs/nhis/about_nhis.htm

[18] CDC. Behavioral risk factor surveillance system (cited 2014 April 21). http://www.cdc.gov/brfss/about/about_brfss.htm

[19] Miller JW, Plescia M, Ekwueme DU (2014). Public health national approach to reducing breast and cervical cancer disparities. Cancer.

[20] Yancy B, DeGroff A, Royalty J, Marroulis S, Mattingly C, Benard VB (2014). Using data to effectively manage a national screening program. Cancer.

[21] U.S. Preventive Services Task Force (2003). Recommendations and rationale screening for cervical cancer: recommendations and rationale. Am Fam Physician.

[22] Tangka FKL, Dalaker J, Chattopadhyay SK, Gardner JG, Royalty J, Hall IJ, DeGroff A, Blackman DK, Coates RJ (2006). Meeting the mammography screening needs of underserved women: the performance of the National Breast and Cervical Cancer Early Detection Program in 2002–2003. Cancer Causes Control.

[23] Corcoran J, Crowley M (2014). Latinas’ attitudes about cervical cancer prevention: a meta-synthesis. J Cult Divers.

[24] Fayanju OM, Kraenzle S, Drake BF, Oka M, Goodman MS (2014) Perceived barriers to mammography among underserved women in a Breast Health Center Outreach Program. Am J Surg 208(3):425–434 10.1016/j.amjsurg.2014.03.005 PMC4135000 24908357

[25] Henry KA, McDonald K, Sherman R, Kinney AY, Stroup AM (2014) Association between individual and geographic factors and nonadherence to mammography screening guidelines. J Womens Health 23(8):664–674 10.1089/jwh.2013.4668 PMC4129969 24865409

[26] Fang DM, Baker DL (2013). Barriers and facilitators of cervical cancer screening among women of Hmong origin. J Health Care Poor Underserved.

[27] CDC (2012). Breast cancer screening among adult women—behavioral Risk Factor Surveillance System, United States, 2010. MMWR.

[28] Ackerson K, Gretebeck K (2007). Factors influencing cancer screening practices of underserved women. J Am Acad Nurse Pract.

[29] Song L, Fletcher R (1998). Breast cancer rescreening in low-income women. Am J Prev Med.

[30] Hall IJ, Rim SH, Johnson-Turbes CA, Vanderpool R, Kamalu NN (2012). The African American women and mass media campaign: a CDC breast cancer screening project. J Womens Health.

[31] Subramanian S, Tangka FKL, Ekwueme DU, Trogdon J, Crouse W, Royalty J (2015) Explaining variation by state in breast and cervical cancer screening proportions in the NBCCEDP (forthcoming, this supplement) 10.1007/s10552-015-0569-5 PMC4748377 25840557

[32] Dalaker J, Proctor BD (2000). U.S. Census Bureau, current population reports, series P60–210, poverty in the United States: 1999.

[33] Pascale J, Rodean J, Leeman J, Cosenza C, Schoua-Glusberg A (2013). Preparing to measure health coverage in federal surveys post-reform: lessons from Massachusetts. Inquiry.

[34] Miller JW, King JB, Ryerson AB, Eheman CR, White MC (2009). Mammography use from 2000 to 2006: state-level trends with corresponding breast cancer incidence rates. Am J Roentgenol.

[35] Ochner MH, Salvail FR, Ford ES, Ajani U (2008). Obesity and self-reported general health, Hawaii BRFSS: are polynesians at higher risk?. Obesity.

[36] Brown ML, Klabunde CN, Cronin KA, White MC, Richardson LC, McNeel TS (2014). Challenges in meeting healthy people 2020 objectives for cancer-related preventive services, National Health Interview Survey, 2008 and 2010. Prev Chronic Dis.

